# Profound Deafness from Accident

**Published:** 1883-07

**Authors:** Robert T. Cooper

**Affiliations:** Physician Diseases of the Ear, London Homœopathic Hospital; London


### PROFOUND DEAFNESS FROM ACCIDENT.

### A CASE THAT ILLUSTRATES THE POWER OF HIGH DILUTIONS.

ROBERT T. COOPER, A. M., M. D., Physician Diseases of the Ear, London Homœopathic Hospital.

Beyond question, whatever may be said against specialism in medicine, it has this great advantage, that it enables the physician to pronounce more authoritatively upon the practicability of curing certain forms of disease than would be possible for those not in every-day contact with similar groups of cases. For this reason the homœopathic specialist is very favorably placed for making trial of the comparative effects of low and of high dilutions. He deals with one form of disease and has ample opportunity of observing to what extent these particular affections are acted upon by the various preparations of our many remedies; hence he is able to make comparisons that under other circumstances would be impossible. In the month of February, 1882, I was consulted about a poor girl, aged twenty, who was absolutely deaf, so much so that it was impossible to hold any conversation with her, and the only history I could get was that four years ago she had had a very severe blow on the side (left) of her head with the handle of a windlass, and that since then she had become gradually deaf, until now she was unable to hear anything.

The appearance of the membranes indicated a very hopeless form of deafness; the left ear was discharging and the membrane perforated, while the membrane of the right ear looked thickened, of a fleshy color, and sunken in, and lying, apparently, against the promontory.

There was no hearing in either ear, for a watch placed in firm contact against the auricle and the vibrations of the tuning-fork could only barely be recognized.

The whole history and aspect of the case was grave in the extreme, and I explained to the lady accompanying her the hopeless nature of the case, adding that the only circumstance in her favor was her age, and that being young it would be only right to afford her the slender chance there was of improving her hearing by treatment.

This opinion was based upon the fact that the deafness resulting from severe injury to the head is invariably a very obstinate form, especially when the injury dates long back and when the power of hearing is so greatly diminished as in her case. In these cases the auditory nerve has lost its function, a certain paralysis of it has occurred, and remedial means are generally powerless to restore it again to activity.

My prescription for her was *Arnica* 30, two pilules three times a day, and an ointment of 15 grs. of *Ungt.-Hydrarg. Nit.* to ½ oz. of *Vaseline* for the discharging ear.*

On August 4th I received the following letter:

"DEAR SIR:—On 9th February last you were kind enough to examine the ears of a poor girl, named ——, and you then expressed a wish to see her again in six months. —— has persevered with your treatment, and has, I am glad to say, derived far more benefit than we dared hope for, as you pronounced her case almost hopeless."

* The use of this ointment mars an interesting case. No reasons for its use are given!—ED. H. P.

On the 18th August I took this note: "Can hear half as well again; hears conversation now, and often can even do without a trumpet. Hears a watch slightly on the left side."

Prescribed *Sulphur* 30, also in pilules.

On 8th November, 1882, I again saw her. The left ear had quite healed and she could hear conversation quite well without a trumpet, and the watch was heard well at three and a half inches on the right and on contact on the left side. Notwithstanding this great improvement, however, she would have it the first pilules (*Arnica* 30) had done her most good, and she volunteered the statement that while before treatment her nervousness was so great as to make it impossible to sleep in a room without a light, that now the darkness is not at all unpleasant.

She can now earn a livelihood, which, without treatment, would have been impossible.

The great lesson we learn from this case, and which one acquainted with the treatment of a very large number of cases of a similar description is in a position to testify to, is this, that it would have been impossible to have effected this change without any but a high dilution.

As for allopathic treatment, there is no allopath who could even believe in the power of medicine to effect such a change as occurred in this case.

Poor Dr. Kitto's account of his deafness, which resulted from accident, is painful in the extreme.* He thus speaks: "Time passed on (after the accident) and I slowly recovered strength, but my deafness continued. The doctors were perplexed by it. They probed and tested my ears in various fashions. The tympanum was uninjured and the organ seemed in every respect perfect, excepting that it would not act. Some thought that a disorganization of the internal mechanism had been produced by the concussion; others that the auditory nerve had been paralyzed.

"They poured into my tortured ears various infusions, hot and cold; they bled me, they blistered me, leeched me, physicked me, and at last they put a watch between my teeth, and, on finding I was unable to distinguish the ticking, they gave me up as a bad case and left me to my fate."

And then, after describing how a seton was inserted in his neck

* *The Lost Senses.* By John Kitto, D. D. London, 1845.

by a subsequent adviser, he goes on to say: "I have learned to acquiesce in it and to mold my habits of life according to the conditions which it (the deafness) imposes, and hence have been unwilling to give footing for hopes and expectations which I feel in my heart can never be realized."

Alas! poor Dr. Kitto. Prejudice prevented your advisers from making trial of a high dilution of *Arnica.—Homœopathic World.*

